# Ethnic differences in staphylococcus aureus acquisition in cystic fibrosis

**DOI:** 10.1016/j.jcf.2023.07.004

**Published:** 2023-07-16

**Authors:** Meghan E. McGarry, Chiung-Yu Huang, Ngoc P. Ly

**Affiliations:** a Division of Pediatric Pulmonology, Department of Pediatrics, University of California, 550 16th Ave, Box 0632, San Francisco, CA 94158, United States; b Department of Epidemiology and Biostatistics, University of California, San Francisco, United States

**Keywords:** Cystic fibrosis, Health disparities, Pulmonary function, Staphylococcus aureus, Hispanic, Latino, Ethnicity

## Abstract

**Background:**

Hispanic people with CF (pwCF) have increased morbidity than non-Hispanic White pwCF, including increased risk of *Pseudomonas aeruginosa*. We aimed to determine if *Staphylococcus aureus (S. aureus)* acquisition varies between Hispanic and non-Hispanic White pwCF.

**Methods:**

This longitudinal cohort study of pwCF ages 0–25 years in the CF Foundation Patient Registry compared acquisition of methicillin-sensitive *S. aureus* (MSSA), methicillin-resistant *S. aureus* (MRSA), persistent MRSA between Hispanic and non-Hispanic White pwCF. Risk of acquisition was assessed by Kaplan-Meier survival curves and its association with ethnicity was evaluated using Cox regressions. Adjusted associations were evaluated using multivariate Cox models adjusting for sex, age of entry into CFFPR, *CFTR* variant severity, pancreatic insufficiency, CF-related diabetes, maternal education, insurance status.

**Results:**

Of 10,640 pwCF, 7.5% were Hispanic and 92.5% were non-Hispanic White. Hispanic pwCF had a 19% higher risk of acquiring MSSA (HR 1.19, 95% CI 1.10–1.28, *p*<0.001) and 13% higher risk of acquiring MRSA (HR 1.13, 95% CI 1.02–1.26, *p* = 0.02) than non-Hispanic White pwCF. The difference in persistent MRSA between ethnicities did not reach statistical significance. After adjusting for confounding variables, only the risk of MSSA was significantly associated with ethnicity. Compared to non-Hispanic White pwCF, Hispanic pwCF acquired MSSA and MRSA at younger median ages (4.9 vs. 3.8 years (*p*<0.001), 22.4 vs. 20.8 years (*p* = 0.02).

**Conclusion:**

Hispanic pwCF <25 years of age have an increased risk of acquiring MSSA and acquired MSSA and MRSA at an earlier age. Differences in *S. aureus* acquisition may contribute to increased morbidity in Hispanic pwCF.

Cystic Fibrosis (CF), an inherited disease that occurs in all races and ethnicities, is not a uniform experience in Hispanic people with CF (pwCF) compared to non-Hispanic White pwCF. Hispanic pwCF have 1.27 to 2.81 times higher risk of mortality and die earlier compared to non-Hispanic White pwCF [1,2]. Hispanic pwCF have lower pulmonary function than non-Hispanic White pwCF with 5.8% lower percent predicted forced expiratory volume in the first second (FEV_1_) overall [3,4]. The difference in pulmonary function between ethnicities is not uniform across the United States and is as wide as 9.0% lower percent predicted FEV_1_ in Hispanic pwCF living in the West [4]. It is not known why Hispanic pwCF have worse pulmonary disease and increased mortality. However, pulmonary infections have been shown to negatively affect morbidity and mortality in CF and also vary by ethnicity. Hispanic pwCF are at increased risk of acquiring *Pseudomonas aeruginosa* and converting to more severe forms (mucoid, chronic, multidrug-resistant) than non-Hispanic White pwCF [5]. Not only are Hispanic pwCF at increased risk of acquiring *Pseudomonas aeruginosa,* but they do so at an earlier age than non-Hispanic White pwCF. Ethnic differences in other pulmonary pathogens have not been investigated.

*Staphylococcus aureus (S. aureus)* has been a known pulmonary pathogen in CF since the first manuscript of CF by Dr. Dorothy Andersen [6]. The prevalence of *S. aureus* has dramatically increased in the CF population included in the CF Foundation Patient Registry (CFFPR) over the past decades: methicillin-resistant *S. aureus* (MRSA) prevalence increased from 2% in 1996 to 25% in 2019, and methicillin-sensitive *S. aureus (MSSA)* prevalence was over 50% in 2019 [7]. MSSA prevalence peaks between 5 and 15 years old, while MRSA prevalence peaks at 15–25 years old. *S. aureus* is associated with increased airway inflammation and increased decline in pulmonary function [8]. MRSA is associated with worse pulmonary function and increased mortality [9, 10]; however, it remains unclear if MRSA is a marker of more severe pulmonary disease or if MRSA negatively impacts the CF disease course [11].

We hypothesize that Hispanic pwCF are at increased risk of *S. aureus* compared to non-Hispanic pwCF. Using a longitudinal pediatric CF study, we sought to determine if the risk and timing of *S. aureus* acquisition vary between Hispanic and non-Hispanic White pwCF in the United States.

These results were previously presented and published as an abstract.

## 1. Methods

### 1.1. Overall design

This is a registry-based cohort study of Hispanic and non-Hispanic White pwCF with longitudinal follow-up to examine ethnic differences in the overall risk of acquisition of *S. aureus* and age at acquisition. We examined incident methicillin-sensitive *S. aureus* (MSSA), incident methicillin-resistant *S. aureus (MRSA),* and persistent MRSA. Respiratory microbiologic cultures were collected when clinically indicated or routinely collected at least quarterly at accredited CF centers.

### 1.2. Study population

The U.S. Cystic Fibrosis Foundation Patient Registry (CFFPR) is a retrospective observational registry study of patients from accredited CF centers, which includes approximately 81–84% of PwCF in the United States [12]. The CFFPR is the largest database of pwCF who are Hispanic. All pwCF included in this study were either Hispanic or non-Hispanic White, <25 years old, diagnosed with CF, and included in the CFFPR between 2008 and 2013.

The primary predictor was self-identified race and ethnicity, defined as Hispanic or non-Hispanic White. Hispanic subjects of all races were included. Other races and ethnicities were excluded.

The primary outcome was the age at the first positive culture (acquisition) of *S. aureus* respiratory infection: incident MSSA, incident MRSA, and transition from intermittent to persistent MRSA. Persistent MRSA was defined as three or more positive respiratory cultures for MRSA during follow-up [13]. The time of development of persistent MRSA was determined as the time of the third culture positive for MRSA.

### 1.3. Statistical methods

We included all data in the CFFPR for each pwCF from date of entry into CFFPR until either 2018 or age 25 years. The events of interest are incidence of *S. aureus* (MSSA, MRSA) and frequency of transition from intermittent to persistent MRSA. To assess the risk of acquisition over age, a separate time-to-event analysis was performed for each form of *S. aureus* where the time origin was set to be the date of birth and the censoring time was defined as the last CFFPR encounter or 25 years of age. Data was truncated at 25 years of age as prior research has found MRSA was associated with pulmonary function decline in children, but not adults, with CF [13]; we are specifically interested in early life factors that potentially contribute to the ethnic disparity in pulmonary function. The median age of initial acquisition for each form of *S. aureus* was summarized using the Kaplan-Meier method and compared between ethnicities using the log-rank test. In addition, the event-free probabilities at 2.5, 5, and 10 years of age were compared between Hispanic and non-Hispanic White pwCF using the Wald-type tests. Cox’s proportional hazards models were used to evaluate the difference in risk for acquiring each form of *S. aureus* between ethnicities. Two multivariate Cox regressions models were further adjusted for covariates that were chosen *a priori* based on prior literature that may be associated with ethnicity and/or *S. aureus* acquisition. Model 1 included the following clinical variables: sex, age of entry into CFFPR, *CFTR* variant class severity (Class I-III, Class IV-V, Unclassified), pancreatic insufficiency (pancreatic enzyme replacement therapy use or none), CF-related diabetes. Model 2 included the variables in Model 1 plus socioeconomic status variables: maternal education (missing, no college vs. any college) and insurance status (private insurance, public insurance, vs. no insurance). Age of entry into CFFPR was chosen as it accounts for the fact that those who enrolled at an older age will have an older age at first positive *S. aureus* test after entering the CFFPR. The following covariates were entered as time-dependent covariates: pancreatic insufficiency, CF-related diabetes, and insurance status. To assess the proportional hazards assumption, we conducted the Schoenfeld test and plotted Schoenfeld residuals for each predictor. The proportional hazards assumption held for all covariates, except for age at enrollment. The quadratic form of age at enrollment was included in the Cox model and as our findings were unchanged, we used the simpler model.

Ethnic differences in clinical characteristics and demographics at the time of study entry were compared using Chi-squared tests for categorical variables and Student’s *t* tests for continuous variables. A 2-sided P-value <0.05 was considered statistically significant. Statistical analysis was performed in R 3.6.2 (R Core Team 2019). The study was approved by the University of California, San Francisco Institutional Review Board (15–17,491) and the CF Foundation Registry Comparative Effectiveness Research Committee.

## 2. Results

### 2.1. Study population characteristics

Of 10,640 pwCF in the CFFPR and under 25 years old, 797 (7.5%) were Hispanic and 9843 (92.5%) were non-Hispanic White (Table 1). There were no statistically significant differences in sex, CF-related diabetes, or the age of enrollment into the CFFPR. Pancreatic sufficiency was slightly less prevalent in Hispanic pwCF than non-Hispanic White pwCF (95.0% vs. 96.5%). Hispanic pwCF were less likely than non-Hispanic White pwCF to have a maternal college education (47.9% vs. 64.1%) and private insurance (43.2% vs. 59.4%). Hispanic pwCF are more likely to have mild *CFTR* variants (Class IV-V) or unknown/unclassified *CFTR* variants.

### 2.2. Respiratory cultures

Hispanic pwCF had slightly more cultures annually than non-Hispanic White pwCF (median 3.0 vs. 2.6, *p*<0.001). The first respiratory culture was done at a slightly younger age in Hispanic pwCF compared to non-Hispanic pwCF (median 1.4 vs. 1.6 years old, *p* = 0.04). Hispanic pwCF had a shorter median follow-up time compared to non-Hispanic White pwCF with all data truncated after 25 years old (17.9 vs. 21.8 years, *p*<0.001). A similar proportion of cultures were from the upper airway (posterior pharyngeal culture) in Hispanic pwCF and non-Hispanic pwCF (43.9% vs. 43.1%, *p* = 0.5).

### 2.3. Age at acquisition

Hispanic pwCF acquired MSSA (median 3.8 vs. 4.9 years, *p*<0.001) and MRSA (median 20.8 vs. 22.4 years, *p* = 0.02) at a younger age than non-Hispanic White pwCF based on the estimated Kaplan-Meier curves. The median age of developing persistent MRSA cannot be determined as neither group reached 50% of pwCF developing persistent MRSA.

### 2.4. Risk of acquiring staphylococcus aureus

**Fig. 1.**
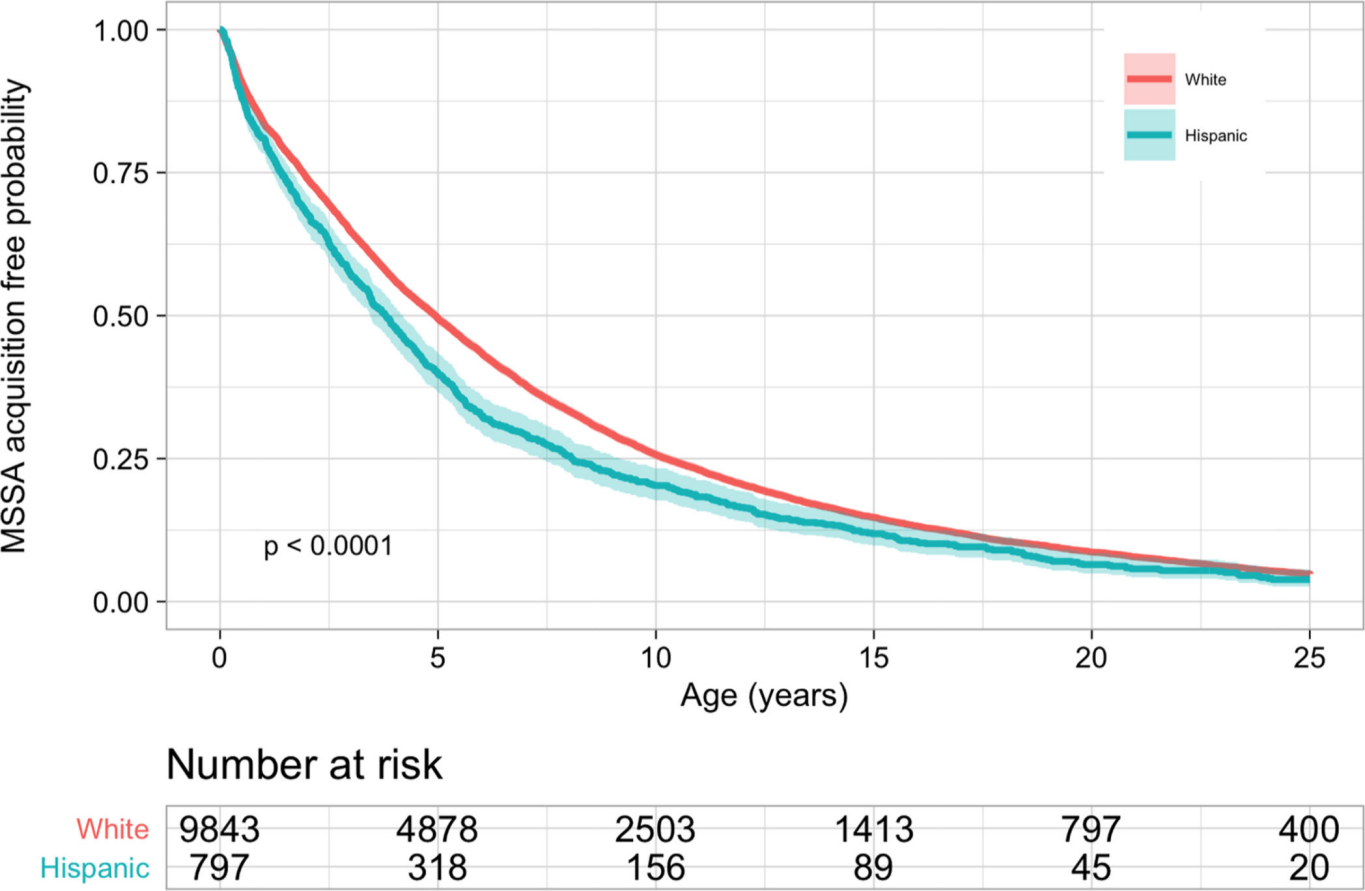
Kaplan-Meier Curves for the Risk of Methicillin-Sensitive *S. aureus* By Ethnicity Hispanic pwCF acquired MSSA at 3.8 years old compared to 4.9 years old in non-Hispanic White pwCF. Hispanic pwCF had a 19% (HR 1.19 95% CI 1.10–1.28, *p*<0.001) higher risk of acquiring MSSA than non-Hispanic white pwCF. The x-axis is age in years. The y-axis is the probability of not having MSSA.

**Fig. 2.**
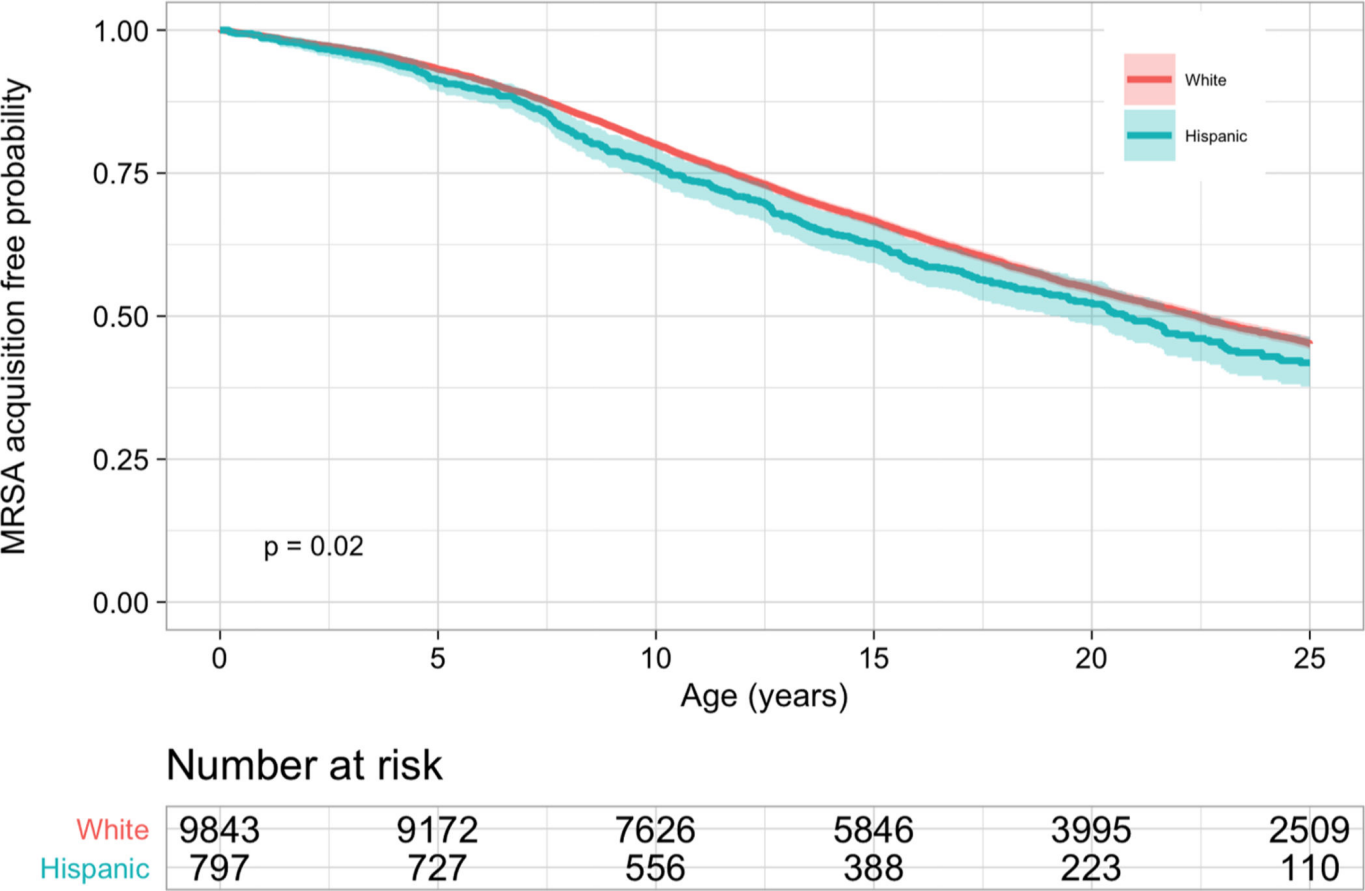
Kaplan-Meier Curves for the Risk of Methicillin-Resistant *S. aureus* By Ethnicity Hispanic pwCF acquired MRSA at 20.8 years old compared to 22.4 years old in non-Hispanic White pwCF. Hispanic pwCF had a 13% (HR 1.13, 95% CI 1.02–1.26, *p* = 0.2) higher risk of acquiring MRSA than non-Hispanic White pwCF. The x-axis is age in years. The y-axis is the probability of not having MRSA.

**Fig. 3.**
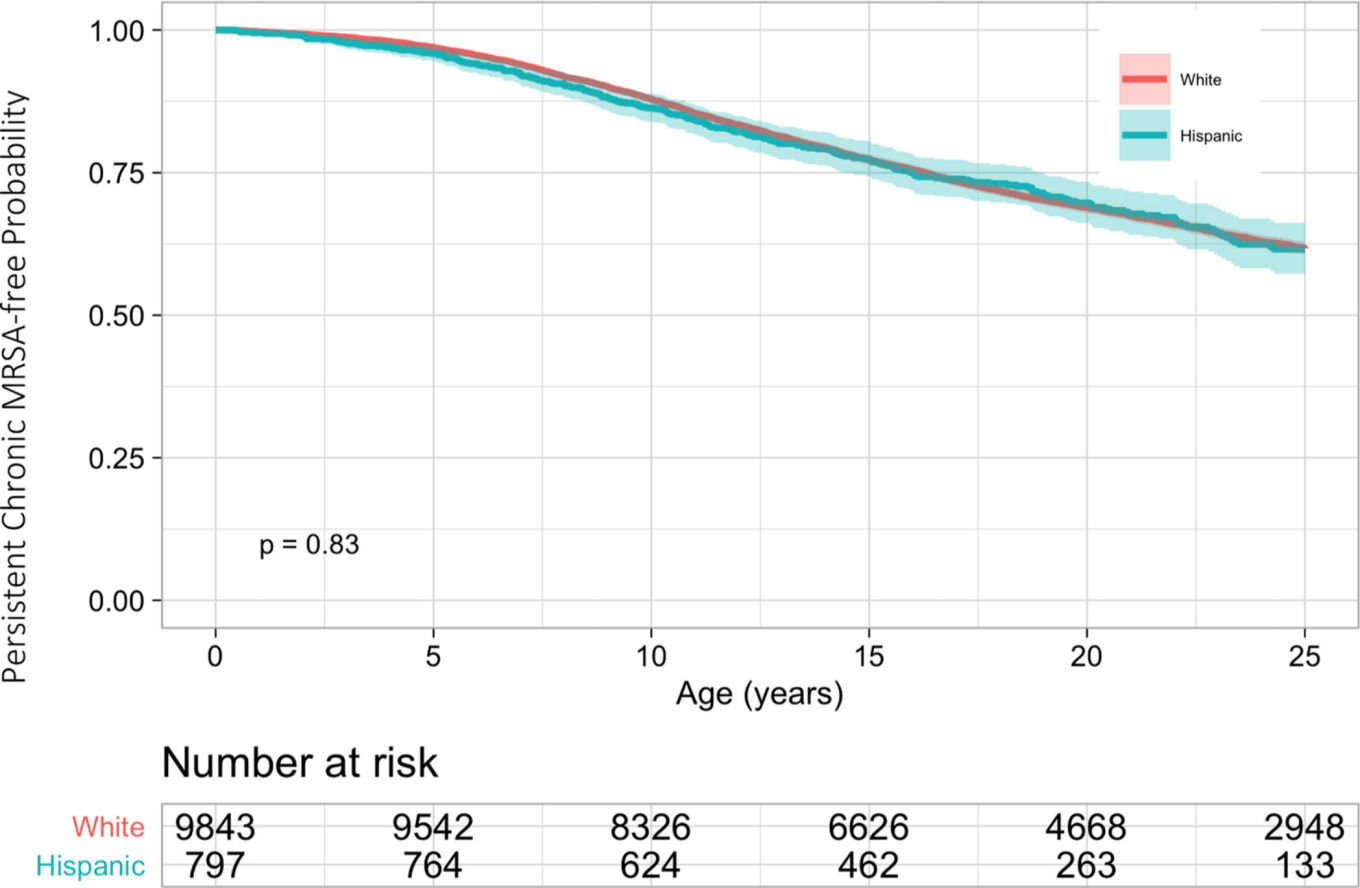
Kaplan-Meier Curves for the Risk of Persistent Methicillin-Resistant *S. aureus* By Ethnicity There was no significant difference in acquiring persistent MRSA (HR 1.01, 95% CI 0.89–1.16, *p* = 0.8) between Hispanic and non-Hispanic White pwCF. The x-axis is age in years. The y-axis is the probability of not having persistent MRSA.

The risk of acquiring *S. aureus* was not uniform between Hispanic and non-Hispanic White pwCF (Figs. 1–3). Compared to non-Hispanic White pwCF, Hispanic pwCF had a 1.19 times higher risk of acquiring MSSA (HR 1.19, 95% CI 1.10–1.28, *p*<0.001) and 1.13 times higher risk of acquiring MRSA (HR 1.13, 95% CI 1.02–1.26, *p* = 0.02) in univariate analyses (Table 2). There was no statistically significant difference in developing persistent MRSA (HR 1.01, 95% CI 0.89–1.16, *p* = 0.8) between Hispanic and non-Hispanic White pwCF in univariate analysis.

With the inclusion of clinical and biological variables in Model 1, Hispanic pwCF had a 1.21 times higher risk of acquiring MSSA (HR 1.21, 1.12–1.30, *p*<0.001) and a 1.10 times higher risk of acquiring MRSA (HR 1.10, 0.99–1.23, *p* = 0.08) than non-Hispanic White pwCF. When adjusted for SES variables as well as clinical and biological variables (Model 2), the risk of MSSA was 1.18 times higher in Hispanic pwCF (HR 1.18, 1.09–1.27, *p*<0.001)) compared to non-Hispanic White pwCF; the risk of MRSA was not different between groups (HR 1.03, 0.93–1.15, *p* = 0.5). There was no statistically significant difference in risk of developing persistent MRSA between Hispanic and non-Hispanic White pwCF when adjusted for clinical and biological variables in Model 1 (0.98, 0.86–1.12, *p* = 0.08). Hispanic pwCF were at 0.90 decreased risk of developing persistent MRSA than non-Hispanic White pwCF, although not reaching statistical significance (HR 0.90, 0.78–1.03, *p* = 0.1).

There was a statistically significant difference in the risk of acquiring MSSA at the age of 2.5 years old and a statistically significant difference in the risk of acquiring MRSA at the age of 10 years old between ethnicities (Table 3). There was not a statistically significant difference in the probability of developing persistent MRSA at the ages 2.5, 5, or 10 years old between ethnicities.

## 3. Discussion

Our pediatric and young adult cohort study using the largest database of Hispanic pwCF comprehensively investigated differences in respiratory infection with *S. aureus* between Hispanic and non-Hispanic White pwCF. Hispanic pwCF suffer an increased burden from CF, most notably more severe pulmonary disease and higher mortality [1–4]. As respiratory infections negatively contribute to morbidity and mortality with CF and we previously described that Hispanic pwCF had an increased risk of *P. aeruginosa* pulmonary infection [5], we had hypothesized that Hispanic pwCF would be at increased risk of acquiring *S. aureus* overall and acquiring it at a younger age compared to non-Hispanic pwCF.

In this large cohort of children and young adults with CF, we found that Hispanic pwCF acquired *S. aureus* at a younger age than non-Hispanic White pwCF. Hispanic pwCF acquired MSSA 1.1 years earlier and MRSA 0.6 years earlier than non-Hispanic White pwCF. The difference in MSSA occurred early in life, as there was a statistically significant lower risk of MSSA at 2.5 years old in Hispanic pwCF, which is before pulmonary function could be measured. These are significant findings as it means Hispanic pwCF have more years of *S. aureus* exposure and, in turn, more years of chronic inflammation [14]. Chronic pulmonary inflammation in CF leads to structural lung damage such as bronchiectasis and lower pulmonary function even early in life [8]. Acquiring *S. aureus* years earlier, even if just intermittently, may contribute to more severe pulmonary function by age six years and higher mortality in Hispanic pwCF.

Our analyses support previous reports that the acquisition of pulmonary pathogens in CF, such as *P. aeruginosa, Burkholderia* complex, and allergic bronchial pulmonary aspergillosis, is not uniform amongst racial and ethnic groups [2,5,15]. Besides the earlier age of acquisition of *S. aureus*, we found that Hispanic pwCF had a higher risk of acquiring MSSA in univariate analysis and multivariate models with the inclusion of potentially confounding clinical/biological factors and socioeconomic factors than non-Hispanic White pwCF. Although there was an increased risk of MRSA acquisition in Hispanic pwCF, this risk was no longer statistically significant after adjusting for clinical and biological factors (sex, pancreatic sufficiency, *CFTR* variant severity, age enrolled in CFFPR, CFRD) and socioeconomic factors (insurance status and maternal education). Despite Hispanic pwCF having a higher risk of MSSA and MRSA transiently, there was no statistically significant difference in the development of persistent MRSA in either univariate analysis or with adjustment for potential confounders.

Our study in the first longitudinal investigation of ethnic differences in S. aureus in pwCF to our knowledge. Using 2010 CFFPR data, Rho et al. found a lower prevalence of MRSA in univariate analyses, as only 17.5% of Hispanic PwCF had MRSA compared to 24.6% of non-White Hispanic pwCF [2]. They did not examine the difference in MSSA. Kopp et al. did not find a difference in MSSA or MRSA prevalence in either Hispanic or African American pwCF compared to the entire population in a cross-sectional analysis of CFFPR data from 2007 to 2012 [16]. Psoter et al. found no difference in MSSA prevalence by ethnicity, and Hispanic pwCF were less likely to have MRSA compared to non-Hispanic PwCF in univariate analysis [17].

Our findings in pwCF differ from those of *S. aureus* in the non-CF population. In the general U.S population, Hispanic people have a lower risk of MSSA and MRSA colonization than non-Hispanic White people [18–20]. There was also a lower risk of MRSA colonization in the general pediatric Hispanic population [21]. In contrast, we instead found an increased risk of MSSA and MRSA in Hispanic pwCF. The reasons for this difference are unknown, but may include increased risk for false negative newborn screening, delayed diagnosis, under-treatment, and environmental exposures [22]. Although Hispanic cwCF had slightly more cultures annually, the difference in culture frequency was minimal and is unlikely to explain the years difference in acquisition between Hispanic and non-Hispanic white cwCF.

Respiratory *S. aureus* in infancy was associated with an increased decline in pulmonary function compared to no *S. aureus* [8]. *S. aureus* was associated with increased pulmonary inflammation, including neutrophil elastase, in bronchoalveolar lavage samples [23–25]. While it has been described that MSSA is associated with less severe obstructed lung disease than MRSA in pwCF [10], there is limited information about the impact of MSSA on pulmonary function or mortality compared to those without any *S. aureus*. MRSA is associated with more severe pulmonary disease, including lower FEV_1_ percent predicted in both children and adults with CF [10,13] and a faster rate of decline in FEV_1_ percent predicted in children and young adults with CF [13]. A prior study did not find an association of MRSA and lung function decline in adults with CF [9]. Decline in FEV_1_ percent predicted, but not baseline lung function, was a risk factor for persistent MRSA [26]. There is debate whether MRSA is a marker of more severe lung disease rather than directly contributing to more severe lung disease [11]. Despite whether MRSA is a marker or causation of severe pulmonary disease, it was not surprising to find an increased risk of MRSA in Hispanic pwCF since they have more severe pulmonary disease than non-Hispanic White pwCF. Unfortunately, previous studies of the effect of MSSA, MRSA, and persistent MRSA on pulmonary function in CF did not include race or ethnicity in their analyses. Further studies are needed to understand how MSSA and MRSA independently contribute to the observed disparities in pulmonary function in Hispanic pwCF. Although we did not compare S. aureus acquisition in Hispanic cwCF to Black cwCF, further studies of how acquisition of respiratory pathogens, such as *S. aureus* or *Pseudomonas aeruginosa*, vary in Black cwCF are needed.

### 3.1. Limitations

One of the limitations of our study is that respiratory cultures were done in clinical practice; therefore, data was not collected at fixed intervals, as this was observational data from the CFFPR. There were slightly more cultures done annually in Hispanic pwCF compared to non-Hispanic White pwCF, which could lead to earlier detection in Hispanic pwCF. However, the difference in culture timing was minimal, while we found the difference in age of acquisition was years earlier in Hispanic pwCF. The sensitivity and specificity for *S .aureus* isolates vary by sample type: upper airway (posterior pharyngeal culture) or the lower airways (bronchoalveolar lavage, sputum). Overall, the specificity of upper airway cultures predicting lower airway pathogens in CF is higher than sensitivity [27,28]. A similar proportion of culture samples were from the upper airway in Hispanic and non-Hispanic White pwCF. Since our findings are based on the U.S CFFPR data, our findings may not apply to Hispanic pwCF residing in other countries besides the U.S..

Our findings may not be generalizable to current differences in *S. aureus* acquisition as we used data from the CFFPR prior to the widespread use of CFTR modulators. CFTR dysfunction leads to inflammation and pulmonary infections. With correction of CFTR dysfunction with CFTR modulators, studies have shown fewer positive cultures [29], slower time to acquiring new pathogens [30], and decreased risk of acquiring new infections [31]. However, it does not appear that CFTR modulators are associated with clearing pulmonary infections [30]. As Hispanic pwCF are less likely to qualify for CFTR modulators than non-Hispanic White pwCF [32], we would expect that there would be a more considerable difference in *S. aureus* than the findings of our study if CFTR modulators slow or reduce *S. aureus* acquisition.

One limitation is that we could not examine ethnic differences in small-colony variants (SCVs) *S. aureus* as few CF centers test for SCVs and are not recorded in the CFFPR. *S. aureus* SCVs are associated with reduced pulmonary function in children with CF [33]. One prior study describing the prevalence of *S. aureus* SCVs in children with CF did not find a difference in prevalence in Hispanic versus non-Hispanic White children; however, small numbers of Hispanic children were enrolled in the study [34]. Further studies examining ethnic differences in *S. aureus* SCVs are needed to understand whether they may contribute to severe lung disease in Hispanic pwCF.

In conclusion, to determine differences in timing and risk of *S. aureus* acquisition in Hispanic pwCF, we longitudinally analyzed over 10,000 people with CF. We found that Hispanic pwCF acquires MSSA and MRSA at an earlier age and are at increased overall risk of acquiring MSSA and MRSA but not developing persistent MRSA. Ethnic differences in pulmonary pathogens may contribute to increased morbidity and mortality in Hispanic pwCF. These results add to the evidence that the disease course in Hispanic pwCF is more severe than in non-Hispanic White pwCF.

## Figures and Tables

**Table 1:** Subject characteristics.

	Hispanic (*n* = 797)	Non-Hispanic White (*n* = 9843)	p-value

Sex, female (% female)	376 (47.2%)	4595 (46.7%)	0.8
Pancreatic Insufficiency (%)	757 (95.0%)	9537 (96.9%)	0.005
*CFTR* Variant Class (%)			0.02
Class I-III	475 (59.6%)	7868 (79.9%)	
Class IV-V	82 (10.3%)	639 (6.5%)	
Unknown	240 (30.1%)	1336 (13.6%)	
Enrollment Age, years median (range)	0.79 (0–24.7)	0.96 (0–25.0)	0.08
CF-Related Diabetes (%)	249 (31.2%)	2189 (32.4%)	0.5
Maternal Education (%)			<0.001
College or more	382 (47.9%)	6310 (64.1%)	
High School or less	294 (36.9%)	1957 (19.9%)	
Missing	121 (15.2%)	1576 (16.0%)	
Insurance			<0.001
Public	444 (55.8%)	3920 (39.8%)	
Private	344 (43.2%)	5851 (59.4%)	
None	8 (1.0%)	71 (0.7%)	

Table 1 includes a description of variables at baseline. Pancreatic insufficiency, CF-related diabetes, and insurance status were included as time-dependent variables in the analyses. Pancreatic insufficiency variable changed in 5.0% of Hispanic subjects and 3.0% of non-Hispanic white subjects. CF-related diabetes variable changed in 30.6% of Hispanic subjects and 31.9% of non-Hispanic subjects. Insurance status variable changed in 59.5% of Hispanic subjects and 61.3% of non-Hispanic subjects.

**Table 2:** Univariate and multivariate analysis of risk of acquiring staph. Aureus by ethnicity.

	MSSA			MRSA			Persistent MRSA		
	Univariate	Clinical & Biological	Clinical, Biological, & SES	Univariate	Clinical & Biological	Clinical, Biological, & SES	Univariate	Clinical & Biological	Clinical, Biological, & SES

**Race & Ethnicity**	1.19	1.21	1.18	1.13	1.10	1.03	1.01	0.98	0.90
	(1.10–1.28)	(1.12–1.30)	(1.09–1.27)	(1.02–1.26)	(0.99–1.23)	(0.93–1.15)	(0.89–1.16)	(0.86–1.12)	(0.78–1.03)
	*P*<0.001	*P*<0.001	*P*<0.001	*P* = 0.02	*P* = 0.08	*P* = 0.5	*P* = 0.8	*P* = 0.8	*P* = 0.1
**Sex**	—	1.03 (0.99–1.07)	1.02 (0.98–1.06)	—	1.12 (1.06–1.18)	1.11 (1.05–1.18)	—	1.17 (1.09–1.25)	1.17 (1.09–1.25)
**Pancreatic**	—	1.40	1.45	—	1.34	1.35	—	1.02	1.35
**Insufficiency**		(1.23–1.60)	(1.27–1.65)		(1.14–1.58)	(1.15–1.58)		(0.95–1.10)	(1.12–1.64)
**CFTR Variant**	—	Ref.	Ref.	—	Ref.	Ref.	—	Ref.	Ref.
**Class I-III**									
**CFTR Variant**	—	1.05	1.05	—	0.84	0.85	—	0.77	0.78
**Class IV-V**		(0.97–1.14)	(0.97–1.14)		(0.73–0.97)	(0.74–0.98)		(0.64–0.93)	(0.65–0.94)
**CFTR Variant**	—	0.97	0.97	—	1.00	1.00	—	1.00	1.00
**Unknown**		(0.92–1.03)	(0.92–1.03)		(0.92–1.09)	(0.92–1.08)		(0.91–1.11)	(0.91–1.11)
**Age Enrolled**	—	0.86	0.87	—	0.85	0.85	—	0.83	0.84
		(0.85–0.87)	(0.86–0.87)		(0.84–0.85)	(0.84–0.86)		(0.82–0.84)	(0.83–0.85)
**CF-Related**	—	1.11	1.10	—	0.98	0.96	—	1.02	0.99
**Diabetes**		(1.05–1.17)	(1.05–1.16)		(0.92–1.04)	(0.90–1.02)		(0.95–1.10)	(0.92–1.07)
**Insurance-Private**	—	—	Ref.	—	—	Ref.	—	—	Ref.
**Insurance- Public**	—	—	1.13	—	—	1.33	—	—	1.54
			(1.08–1.18)			(1.26–1.41)			(1.44–1.65)
**No insurance**	—	—	0.91	—	—	0.69	—	—	1.10
			(0.73–1.12)			(0.49–0.98)			(0.77–1.58)
**Maternal**	—	—	Ref.	—	—	Ref.	—	—	Ref.
**Education High**									
**School**									
**Maternal**	—	—	0.96	—	—	0.97	—	—	0.96
**Education**			(0.92–1.01)			(0.91–1.04)			(0.89–1.04)
**College**									
**Maternal**	—	—	0.92	—	—	0.80	—	—	0.77
**Education**			(0.86–0.98)			(0.72–0.89)			(0.67–0.88)
**Missing**									

**Table 3:** Probability of *S. aureus* Free at 2.5, 5, & 10 years old.

	Non-Hispanic White	Hispanic	p-value

**2.5 years old**			
MSSA	69.4% (68.5–70.3%)	62.6% (59.3–66.1%)	<0.001
MRSA	97.2% (96.9–97.5%)	96.6% (95.4–97.9%)	0.4
Persistent MRSA	99.9% (98.8–99.2%)	98.4% (97.5–99.3%)	0.2
**5 years old**			
MSSA	49.4% (48.5–50.4%)	39.8% (36.5–43.3%)	<0.001
MRSA	93.2% (92.7–93.7%)	91.2% (89.3–93.2%)	0.06
Persistent MRSA	96.9% (96.6–97.3%)	95.9% (94.5–97.3%)	0.1
**10 years old**			
MSSA	25.7% (24.8–26.5%)	20.3% (17.7–23.3%)	<0.001
MRSA	80.0% (79.2–80.8%)	76.3% (73.3–79.4%)	0.02
Persistent MRSA	87.9% (87.2–88.5%)	86.3% (83.9–88.8%)	0.2

P-value is based on Ward-type test at the specific time periods 2.5, 5, 10 years old.

## Abbreviations:

CF: cystic fibrosis
CFPPR: CF Foundation Patient Registry
CFTR: cystic fibrosis transmembrane receptor
FEV_1_: forced expiratory volume in the first second
MRSA: methicillin-resistant staphylococcus aureus
MSSA: methicillin-resistant staphylococcus **▒**aureus
PwCF: people with CF
SCVs: small-colony variants
SES: socioeconomic status
